# Assessment by miRNA microarray of an autologous cancer antigen‐pulsed adoptive immune ensemble cell therapy (AC‐ACT) approach; demonstrated induction of anti‐oncogenic and anti‐PD‐L1 miRNAs

**DOI:** 10.1002/ccr3.2343

**Published:** 2019-09-30

**Authors:** Masanobu Chinami, Kaoru Iwabuchi, Yoshiteru Muto, Yasuhiko Uchida, Ryu Arita, Rana A. Shuraim, Chaker N. Adra

**Affiliations:** ^1^ BFSR Institute Fukuoka Japan; ^2^ The Research Institute of Health Rehabilitation of Tokyo Tokyo Japan; ^3^ Fukuoka MSC Medical Clinics Fukuoka Japan; ^4^ The Adra Institute Boston, MA USA

**Keywords:** adoptive cell therapy, cancer, immunotherapy, miRNA analysis, PD‐1

## Abstract

A 60‐year‐old woman with stage IV rectal cancer received adoptive cell therapy with autologous cancer antigen (AC‐ACT) causing induction of anti‐oncogenic and anti‐PD‐L1 miRNAs as assessed by miRNA microarray. More than 1 year after AC‐ACT, metastases have been arrested, and the patient reports good quality of life.

## Graphical abstract

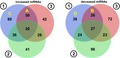

## 1 INTRODUCTION

A rectal cancer patient (stage IV) was treated with an autologous cancer antigen‐pulsed adoptive immune ensemble cell therapy (AC‐ACT) and assessed by miRNA microarray. Tumor‐suppressive miRNAs for colorectal cancer and the PD‐L1 immune checkpoint blocker increased, while tumor‐promotive miRNAs decreased.

Three standard cancer therapies, surgery, chemotherapy and radiotherapy, have been established for multiple decades and continued to be developed.^1^, ^2^ Recently, a fourth therapy, immunotherapy, has emerged into the therapeutic arena.^1^, ^2^ In vivo administration of anticancer agents, even immune checkpoint blockers, give problematic side effects.^3^ In contrast, ex vivo administration via adoptive cell therapy (ACT) gives reduced adverse effects.^4^ Adoptive immune cells therapies (ACT) have been performed extensively (>10 years), at many university hospitals and private clinics in Japan, and on more than 5000 patients with cancer including end‐stage patients who had exhausted standard therapies. Adoptive immune cell therapies have fairly good performance of therapeutic results in comparison with standard therapies. In this case report, we describe a further improvement of this method by adding autologous antigens to the ACT and using miRNA to assess impact.

Most advanced stage of patients with cancer are suppressed in their antitumor immunity and eventually develop a cachexia, with lymphopenia, fatigue, anorexia, loss of adipose, and muscle tissue. In cancer immunotherapy, T cells, NK cells, NKT cells, DC cells, and others are used for ACT.^5^ Vaccination with Wilm's tumor peptide 1 (WT1) for patients with cancer is also widely used.^6^ However, WT1 and other major cancer peptides remain of limited utility for personalized cancer vaccines due to neo‐antigens produced by mutations revealed by NGS analysis.^7^, ^8^ Thus, highly specific tumor antigens for individual patients are a necessary for therapeutic advance. Autologous cancer antigens from surgically excised tumor material may be ideal. These antigens have been used for in vivo vaccination in clinical cases and have improved outcomes.^9^

In contrast to in vivo vaccination, adoptive transfer of immune effector cells is able to modulate immunity ex vivo. This method is a highly personalized cancer therapy that involves administration to the cancer‐bearing host of immune cells with direct anticancer activity. We adopted ex vivo vaccination to dendritic cells (DC) with autologous cancer antigens and cultured them. These DC were then mixed with separately cultured ensemble immune cells containing T, NK, NKT, and other cells (excepting DC), prior to infusion back into a patient with cancer (we term this novel modification of ACT therapy AC‐ACT, autologous cell‐adoptive cell therapy). In a further novel modification of the ACT approach, we assessed outcomes using miRNA microarray analysis at pre‐ and posttherapy. miRNA genes locate intergenic and intragenic noncoding RNA regions in introns or within an exon of the gene. These are suitable markers because they down‐regulate target genes in pathways including cell growth, differentiation, metabolism, and the cell cycle.^10^ Deregulation of miRNAs, both up and down, is found in many cancers. Up‐regulated and down‐regulated miRNAs targeting oncogene and anti‐oncogene mRNAs, respectively, suppress tumor progression.^11^, ^12^

Three patients were selected for AC‐ACT, representing three distinct groups with interest in the AC‐ACT/miRNA approach that we describe here. One is an active patient with cancer (stage IV colorectal cancer). The second and third patients represent emerging classes of patient in Japan, seeking miRNA information on risk or possible early diagnosis while being otherwise healthy, or seeking ACT without autologous cancer antigen as a cancer preventative strategy. For each of these distinct patient types, we present a case report of the protocol used and miRNA outcomes analysis.

## 2 MATERIALS AND METHODS

### 2.1 Patients

#### 2.1.1 Subject 1

**Table:** 

Enrollment criteria:	Active cancer patient with advanced disease and limited clinical options.
Gender:	Female
Age:	58 y
Disease:	Stage IV rectal cancer patient with multiple metastasis (lung, lumbar vertebra, and peritoneum). Received a surgical operation for rectal cancer with stoma before being diagnosed as stage IV in January 2018. At the time of the stage IV diagnosis, patient was inoperable because of lung and vertebral metastasis. AC‐ACT was performed at this stage in disease progression.
Protocol:	Received AC‐ACT and miRNA analysis.

#### 2.1.2 Subject 2

**Table:** 

Enrollment criteria:	Healthy patient desiring miRNA analysis for risk analysis or early diagnosis.
Gender:	Male
Age:	65 y
Disease:	None.
Protocol:	miRNA analysis only.

#### 2.1.3 Subject 3

**Table:** 

Enrollment criteria:	Preliminary diagnosis seeking ACT without autologous cancer antigen as a cancer preventative strategy.
Gender:	Male
Age:	48 y
Disease:	Suspected lung cancer. Tobacco smoker for more than 20 y (20 cigarettes/day) and recently had been coughing for a month. He received medical examinations and showed higher value of a tumor maker, CYFRA (cytokeratin fragment 19, 5.7 ng/mL) and very low values of miR‐154‐5p, let‐7i‐3p, miR‐3202, and miR‐610 by RT‐PCR (data not shown).
Protocol:	ACT without autologous cancer antigen pulse and with miRNA analysis.

### 2.2 Ethical disclosure

Adoptive cell therapy with autologous cancer antigen therapy is approved in Japan. The specific application of this therapy in this study complies with the Declaration of Helsinki and was approved by a Recelling Specific Regeneration Ethics Committee (Osaka, Japan) with the Japan Ministry of Health Protocol Approval Number # PC7180012. In addition, for this therapy involving human subjects, completely informed consent for the three subjects was obtained.

### 2.3 Materials

Antibodies and cytokines, CD3, CD161, IL‐2, IL‐4, GM‐CSF, were purchased from CalBiochem. Ficoll‐Paque was from Sigma.

### 2.4 Processing of cells and culture of NKT and other cells

Ten mL of blood was collected from the subjects. PBMC was separated with Ficoll‐Paque, and a buffy coat fraction was obtained, and CD14 + cells were separated by MACSprep CD14 MicroBeads, human (Miltenyi Biotec) in an ice‐cold MACS buffer. CD14^+^ cells were cultured for dendritic cells. CD14^−^ cells containing T cells, NK cells, NKT cells, and other cells were separately cultured. CD14^−^ cells were cultured in T‐25 flask which had been coated by 1 µg/mL anti‐CD3 antibody (BioLegend) in PBS for overnight (16 hours) and further coated by 10 µg/mL anti‐CD161 antibody (Abgent) in PBS for another overnight period. All the procedures were done in a cell control center approved by National Health Welfare agency.

### 2.5 DC cells and autologous tumor antigen pulse

CD14 + cells containing dendritic cells (DC) were cultured in a complete medium containing 20 ng/mL rhGM‐CSF and 20 ng/mL rhIL‐4 for 4‐5 days in a T‐25 flask. For AC‐ACT, CD14+^+^ cells were pulsed for three hours with autologous cancer antigen (0.1  µg/mL protein) extracted by a kit (Formalin Fixed Paraffin Embedded Protein Isolation Kit, ITSI‐Bioscience) and were used for mixed culture with CD4+^−^ cells. For ACT, CD14+^+^ cells without autologous cancer antigen pulse were used for the mixed culture.

### 2.6 AC‐ACT and ACT therapy

The mixed cells were cultured overnight in the presence of 200 ng/mL α‐GalCer at 37°C in 5% CO2, and 100 mL of AC‐ACT cells (2‐3 × 10^9^) in saline was infused after flowcytometric and aseptic checks. ACT therapy was done in the same manner. Before infusion, the cells were washed three times in saline.

### 2.7 Flow cytometry

A sample of cell (1 × 10^5^ cells) for the infused cells were characterized flow cytometrically by adding PE anti‐Vα24 and APC anti‐CD3 antibody for NKT cells; FITC anti‐CD57 and PE anti‐CD16 antibody for NK cells; FITC anti‐CD4 and PE anti‐CD29 antibody for T cells in 500 μL FACS buffer, and measured on an JSAN FACS machine (BayBio Co.).

### 2.8 miRNA microarray

Paired serums of the two subjects were obtained immediately before and 1 week later of AC‐ACT or ACT. Simultaneously, the paired serum of another control subject 2 was obtained. Following procedures were done by Cell Innovator Co. Each sample of total RNA was prepared from 200 µL serum by miRNeasy Serum/Plasma (Qiagen) The samples (100 ng) were labeled with an miRNA Complete Labeling and Hybridization kit (Agilent, 5190‐0456) according to the Agilent miRNA microarray protocol. The SurePrint G3 Human miRNA kit 80 × 60 k (Human_miRNA_V21.0) array chip was used.

### 2.9 Statistical analysis

In order to examine the effects of AC‐ACT and ACT, the difference of pre‐ and postvalues were calculated. From 2550 miRNAs, top 100‐200 miRNA of values, either increased or decreased, were used. Shared and specific miRNAs among them were analyzed by Venn Diagram (http://bioinformatics.psb.ugent.be/webtools/Venn/).

## 3 RESULTS

### 3.1 Post‐AC‐ACT case history

The patient with cancer, subject 1, who received AC‐ACT, has been free of progression for 1 year after starting AC‐ACT. Metastases have been arrested, and no new metastases have been detected. PET‐CT scans showed that lungs and bone metastasis have been stable and some lesions decreased. The subject reports good quality of life (QOL). Biochemical data from 12 months after stage IV diagnosis and AC‐ACT are as follows: neutrophil/lymphocyte ratio: 1.76, eosinophil: 6.8%; CRP: 0.46 (lowered); total protein: 6.5 g/dL; and Hb: 14.2 g/dL. In addition, AST and ALT indicate normal liver function. Creatinin, BUN, and eGFR show normal kidney function, and plasma electrolytes are normal. Thus, this patient has no anemia, hypoproteinemia, renal, or liver dysfunction, that would be expected in an end‐stage cancer patient in cachexia. We note also that subjects 2 and 3 remain cancer free.

### 3.2 FACS analysis of the ensemble cells

**Figure 1:**
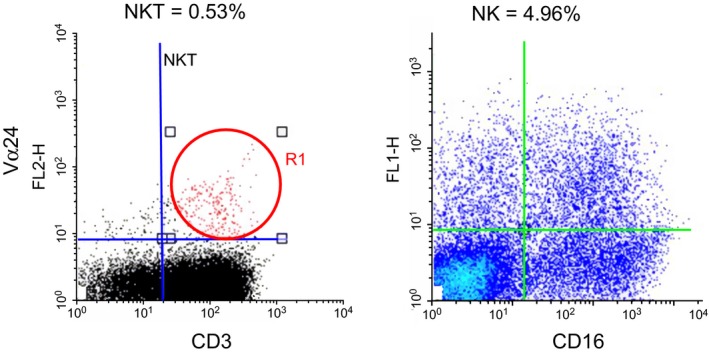
FACS pattern of the infused ACT ensemble cells for subject 3

A typical FACS pattern of the infused cells to subject 2 is shown in Figure 1. NK, NKT, and T‐cell fractions comprised 4.96, 0.53 and 30%, respectively.

### 3.3 Effects of AC‐ACT on oncogenic and anti‐oncogenic miRNA expression

**Figure 2:**
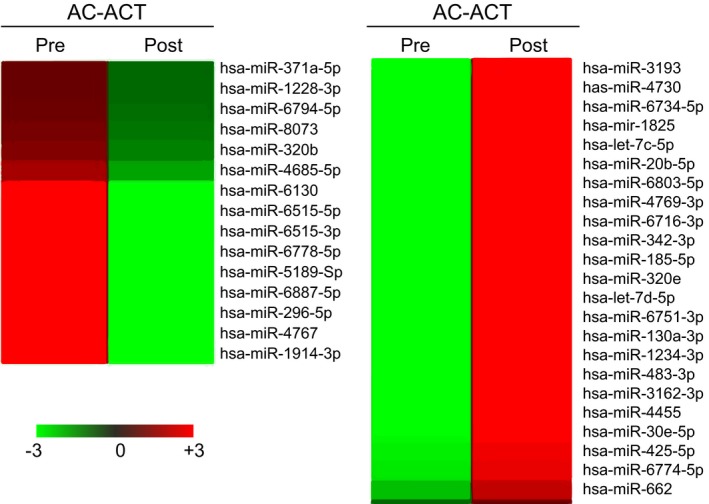
Heatmap of miRNA changes pre‐ and post‐AC‐ACT. miRNA in serum was hybridized with probe fixed on solid chip (Agilent) as described in Methods. Intensity differences between pre‐ and post‐AC‐ACT were clustered: high (red) to low (green)

**Figure 3:**
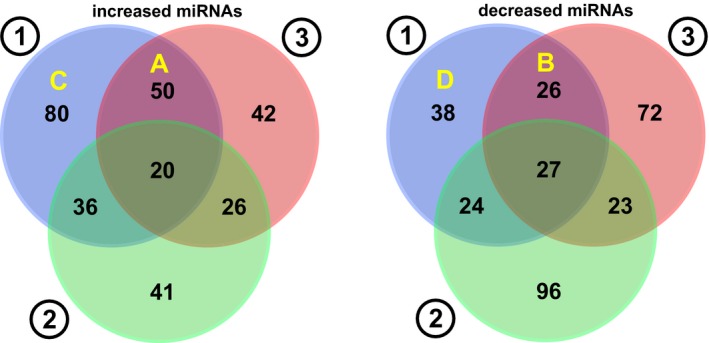
Shared prominently (0.1 cutoff) changed miRNAs by Venn diagram between subjects (1) and (3) after AC‐ACT and ACT, respectively, and those of nontherapy subject (2)

**Figure 4:**
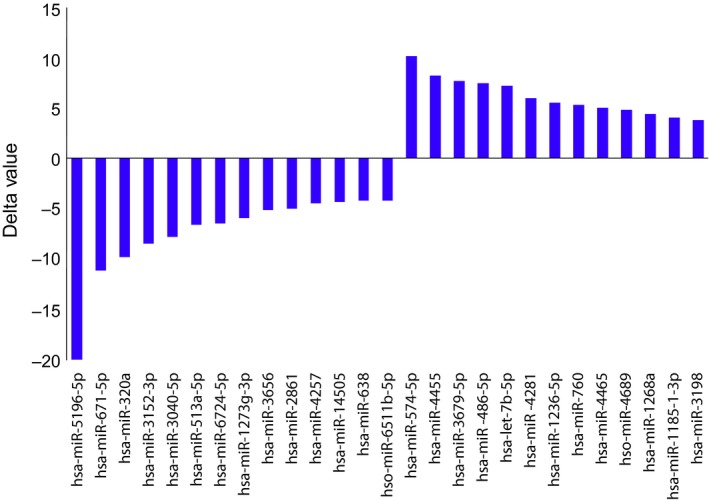
Ordered up/down values shared between subjects 1 and 3 (A and B in Figure 3)

**Figure 5:**
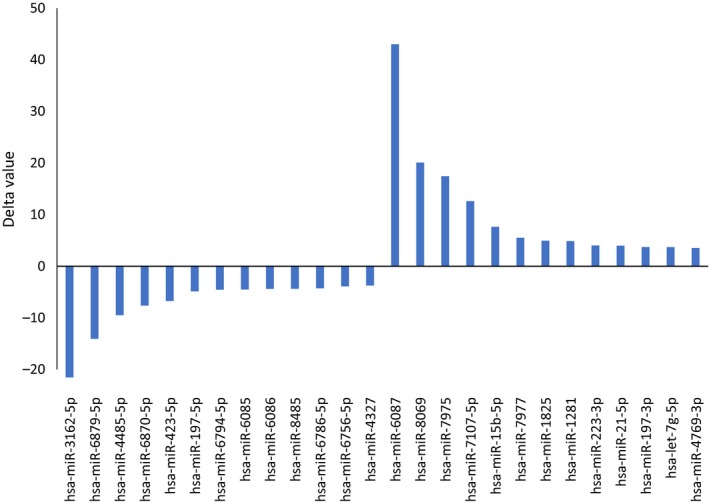
Ordered up/down values specific for subject 1

Paired miRNA microarray examinations were performed for pre‐ and post‐AC‐ACT and ACT for subject 1 and subject 3, respectively, and simultaneously for subject 2 without any ACT. Changes in expressed miRNAs of subject 1 were clustered for largely increased and decreased values as shown in Figure 2. The ordered 100‐200 miRNAs from each subject were selected with a cutoff value, 0.1, and were analyzed by Venn diagram for shared miRNAs among the subjects as shown in Figure 3. The highly, either positive or negative, ordered relative values of miRNAs which were shared in the subjects received AC‐ACT or ACT (areas A and B of subjects 1 and 3, respectively, in Figure 3) are shown in Figure 4. In the same manner, the ordered relative values of specific patient with cancer (areas C and D of subject 1, in Figure 3) are shown in Figure 5. The functions of those miRNAs according to the indicated references are listed in Tables 1, 2.

**Table 1:** Reference‐based tumor suppressor miRNAs either by increasing (targeting oncogenic genes in CRC) or by decreasing (targeting tumor‐suppressive genes in CRC)

AC‐ACT or ACT Received Subjects 1, 3)				Cancer Patient Specific (Subject 1)			
increased	Ref.	decreased	Ref.	increased	Ref.	decreased	Ref.
miR‐574‐5p	25	miR‐671‐5p	38	miR‐6087	47	miR‐3162‐5p	54
miR‐4455	26	miR‐3152‐3p	39	miR‐8069	48	miR‐6879‐5p	45
miR‐3679‐5p	27	miR‐940	40	miR‐7975	49	miR‐6870‐5p	55
miR‐486‐5p	28	miR‐6274‐5p	41	miR‐15b‐5p	50	miR‐423‐5p	56
let‐7b‐5p	29	miR‐1273g‐3p	42	miR‐7977	51	miR‐197‐5p	57
miR‐4281	30	miR‐3656	43	miR‐1281	52	miR‐6794‐5p	59
miR‐1236‐3p	31	miR‐2861	44	miR‐223‐2p	53	miR‐6085	58
miR‐760	32	miR‐4257	45			miR‐8485	59
miR‐4465	33	miR‐4505	46				
miR‐4689	34						
miR‐1268a	35						
miR‐1185‐I‐3p	36						
miR‐3198	37						

Note

Citations for miRNAs described to have tumor‐suppressive effects and that are increased or decreased between pre‐ and post‐AC‐ACT or ACT (shared between subjects 1 and 3: A and B and cancer patient specific one subject 1: C, D in Figure 3). There are two modalities of tumor suppressor miRNAs either by increasing (targeting oncogenes) or by decreasing (targeting tumor suppressor gene).^58^

**Table 2:** Increased values of miRNAs targeting PD‐L1 mRNA after AC‐ACT and ACT

miRNA targeting PD‐1L	Relative fold Increase after AC‐ACT or ACT	
	Subject 1 (AC‐ACT)	Subject 3 (ACT)
miR‐15a‐5p	3.06	1.07
miR‐16‐5p	11.01	56.1
miR‐93‐5p	2.55	3.23

Note

List of miRNAs that show increases after AC‐ACT or ACT, and those target PD‐L1 mRNA.

### 3.4 Effects of AC‐ACT on miRNAs which target an immune checkpoint blocker

The immunosuppressive protein PD‐L1 is up‐regulated in many cancers and lead to poor prognosis.^13^, ^14^ Targeting PD‐L1 is considered as one of therapies for colorectal cancer.^15^, ^16^ To assess the effects of AC‐ACT on miRNAs which target PD‐L1, their levels in pre‐ and post‐AC‐ACT samples were examined. As shown in Table 2, increased levels of miR‐15a‐5p, miR‐16‐5p, miR‐93‐5p, and mR‐106b‐5p, which target PD‐L1, are found in both the subject 1 and subject 3, after AC‐ACT and ACT, respectively.

## 4 DISCUSSION

This paper is a case report on outcomes and a novel miRNA‐based assessment system for the outcomes of an approved therapy that is in widespread use in Japan. There is also an emerging patient‐led desire to use this therapy for cancer prevention. However, AC‐ACT is approved for only patients with cancer by Japanese Health Ministry. Therefore, we sought approval to offer ACT to healthy subjects and assess outcomes by miRNA analysis in order to expand the evidence base for future consideration of its use in monitoring and prevention.

Immunotherapy based on the adoptive transfer of naturally occurring or genetically engineered immune effector cells has therapeutic benefit in clinical trials of advanced cancers. Present AC‐ACT is a combination of DC vaccination with other immune cells. Here, DC cells were induced with IL‐4 and GM‐CSF and were pulsed with autologous cancer antigen. Other mixed immune cells, primarily cultured for NKT cells, a method which was originally developed by Taniguchi,^17^ were also separately induced with IL‐2, αCD3, αCD161, and α‐GalCer. AC‐ACT has potential advantages over the other ACTs such as NK cells only, which are frequently associated with fever (higher than 38°C) as an adverse effect. In contrasts, AC‐ACT is associated with almost no adverse effects after infusion. The patient we presented was largely progression free (assessed by CT examination and quality of life indices) for more than one year, although carcinoembryonic antigen (CEA) gradually elevated from 4.5 to 6.5 ng/mL over this period. In addition to patient outcomes, the current study has implications for rapid, personalized determination of efficacy of ACT therapies by using miRNA array, as markers of progression or response to therapy. In general, it is difficult in cancer therapy to determine the appropriate treatment for each patient, also the costs of therapy are very expensive and so determining the efficacy rapidly is important. Tumor markers such as CEA and Ca‐19‐9 are usually used, but the number of them is limited, and the specificity for certain cancers is ambiguous. The type of miRNA analysis performed here may offer new approaches, but there remain ambiguities and challenges in the use of miRNAs for the follow‐up of cancer status. Because the seed sequences of miRNAs are only ~7 nucleotides, they can target numerous mRNAs, limiting their diagnostic potential by themselves. However, the scale of array analysis (thousands of miRNAs measured simultaneously) leads to large data sets that may be mined for associations and trends. Our results show most that there may be patterns in changed levels of groups of miRNAs that may have utility in tracking progression after therapy. Most changes in miRNA levels that we observed, either increases or decreases, induced by AC‐ACT or ACT, are related to tumor‐suppressive functions according to the literature (Tables 1, 2), suggesting that they potentiate tumor immunity. These types of personalized, individualized medicine approaches where miRNA profiling is monitored for each patient to develop a unique picture of progression after therapy may have more value than attention to normalized or population‐based metrics of therapy effectiveness and progression. Notably, the most significantly decreased miRNA in response to AC‐ACT was miR‐5196‐5p, which targets the Fra2 gene,^18^ plays a critical role in the progression of human cancers,^19^ and it is involved in IL‐4 production for tumor immunity suppressor.^20^ Reduction in miR‐5196‐5p by AC‐ACT may suppress cancer progression but also immune potency will be inhibited. miR‐320a, another prominently decreased by AC‐ACT, suppresses colorectal cancer progression by targeting Rac1,^21^ although high miR‐320a levels appear to induce pro‐tumorigenic M2‐like macrophages.^22^ Low miR‐320a which we observe with AC‐ACT has also been associated with positive efficacy of peptide vaccination for colorectal cancer.^23^ These counteractive functions, cancer suppressive and immune inhibition, are seen for other miRNAs such as miR‐21 and miR‐155 24 and underscore the need for further studies.

## 5 CONCLUSION

The effectiveness of AC‐ACT for colorectal cancer therapy was monitored successfully by miRNA microarray.

## CONFLICT OF INTEREST

The authors indicate that the work was conducted in the absence of any commercial or financial benefits and that there are no potential financial or nonfinancial competing interests..

## AUTHORS' CONTRIBUTIONS

MC and CA: Were responsible for the report conception and design, collection and assembly of data, interpretation, analysis, presentation of data and drafted the manuscript and were responsible for final approval of the manuscript. MC and KI: Performed experiments, such as RNA extraction for microarrays, and were involved in all administrative, experimental, technical, and material support. MC: Was also involved in obtaining the informed consent from all subjects. YM and YU: Provided the medical care to subject 1, the colorectal cancer patient. They were also responsible for obtaining the informed consent from all subjects. RA: Is the owner of the Fukuoka MSC Medical Clinic where AC‐ACT was performed, and he was responsible for the oversight processes including accreditation, licensure, permits, informed consent, and certifications. RS: Assisted in the analysis, interpretation, and presentation of data and also participated in manuscript drafting. All authors read and approved the final manuscript.
